# SREBP-1c-Dependent Metabolic Remodeling of White Adipose Tissue by Caloric Restriction

**DOI:** 10.3390/ijms19113335

**Published:** 2018-10-26

**Authors:** Masaki Kobayashi, Namiki Fujii, Takumi Narita, Yoshikazu Higami

**Affiliations:** 1 Laboratory of Molecular Pathology and Metabolic Disease, Faculty of Pharmaceutical Sciences, Tokyo University of Science, 2641 Yamazaki, Noda, Chiba 278-8510, Japan; kobayashim@rs.tus.ac.jp (M.K.); 3b16710@ed.tus.ac.jp (N.F.); 2 Translational Research Center, Research Institute of Science and Technology, Tokyo University of Science, 2641 Yamazaki, Noda, Chiba 278-8510, Japan; 3 Epidemiology and Prevention Division, Research Center for Cancer Prevention and Screening, National Cancer Center, Tsukiji, Chuo-ku, Tokyo 104-0045, Japan; tanarita@ncc.go.jp

**Keywords:** caloric restriction, white adipose tissue, SREBP-1c, fatty acid synthesis, PGC-1α, mitochondrion

## Abstract

Caloric restriction (CR) delays the onset of many age-related pathophysiological changes and extends lifespan. White adipose tissue (WAT) is not only a major tissue for energy storage, but also an endocrine tissue that secretes various adipokines. Recent reports have demonstrated that alterations in the characteristics of WAT can impact whole-body metabolism and lifespan. Hence, we hypothesized that functional alterations in WAT may play important roles in the beneficial effects of CR. Previously, using microarray analysis of WAT from CR rats, we found that CR enhances fatty acid (FA) biosynthesis, and identified sterol regulatory element-binding protein 1c (SREBP-1c), a master regulator of FA synthesis, as a mediator of CR. These findings were validated by showing that CR failed to upregulate factors involved in FA biosynthesis and to extend longevity in SREBP-1c knockout mice. Furthermore, we revealed that SREBP-1c is implicated in CR-associated mitochondrial activation through the upregulation of peroxisome proliferator-activated receptor γ coactivator-1α (PGC-1α), a master regulator of mitochondrial biogenesis. Notably, these CR-associated phenotypes were observed only in WAT. We conclude that CR induces SREBP-1c-dependent metabolic remodeling, including the enhancement of FA biosynthesis and mitochondrial activation, via PGC-1α in WAT, resulting in beneficial effects.

## 1. Introduction

Caloric restriction (CR), also known as dietary restriction, is a simple and reproducible manipulation that delays the onset of many age-related pathophysiological changes and extends both median and maximum lifespan [1,2]. The life-extending effect of CR is observed in several species, including yeast, worms and mammals; hence, CR has been widely investigated in aging research. In general, CR animals exhibit low body temperature and plasma insulin, and high plasma dehydroepiandrosterone sulfate (DHEAS) [3]. Interestingly, it has been reported that humans with this phenotype live longer than their counterparts [4]. Furthermore, a recent report has revealed the effectiveness of CR in non-human primates, implying that CR can be also beneficial for humans [5]. Previous studies have suggested that the beneficial effects of CR may involve various mechanisms; for example, the suppression of growth hormone/insulin-like growth factor (GH/IGF-1) signaling, reduction of mechanistic target of rapamycin complex 1 activity, activation of sirtuin, enhancement of mitochondrial biogenesis, attenuation of oxidative and other types of stress, suppression of inflammation, and alteration of the gut microbiome [6,7,8]. Moreover, Mitchell and colleagues have demonstrated that CR-associated responses or mechanisms in mice differ according to strain and sex [9]. For example, differences in sex or strain (C57BL/6J or DBA/2J) affect CR-induced changes in metabolism and stress resistance, and sex-specific differences have been identified in mitochondrial architecture and function [9]. Thus, the mechanisms underpinning the effects of CR are complex and diverse, and further research is required for them to be fully elucidated.

White adipose tissue (WAT) is a major site of energy storage in the form of triglyceride (TG), but WAT has also become established as an endocrine tissue that secretes adipokines, such as adiponectin, leptin, monocyte chemotactic protein 1 (MCP1), and tumor necrosis factor α (TNFα). It is accepted that the characteristics of adipocytes and their secretory profile differ according to their size. Large adipocytes storing a large amount of TG, which characterize obesity, secrete less adiponectin and more MCP1 and TNFα, which is associated with inflammation and insulin resistance. In contrast, small adipocytes secrete more adiponectin and less MCP1 and TNFα [10,11,12]. Moreover, small adipocytes are more sensitive to insulin and play a buffering role for whole-body lipids by absorbing them after a meal and releasing them in the fasting state [13]. Thus, differences in the characteristics of WAT can influence whole-body metabolism.

Recent studies have demonstrated that several models of genetic modification in WAT are associated with differences in lifespan. For example, Blüher and colleagues reported that fat-specific insulin receptor knockout (FIRKO) mice display lower adiposity, enhanced mitochondrial biogenesis, and extended lifespan, compared with a control group [14]. In addition, genetic manipulation of master regulators of adipocyte differentiation in mice, including CCAAT enhancer binding protein α (C/EBPα), C/EBPβ, and peroxisome proliferator-activated receptor γ (PPARγ), is known to alter lifespan. Chiu and colleagues demonstrated that mice in which C/EBPα is replaced with C/EBPβ (β/β mice) exhibit lower adiposity and prolonged lifespan [15], whereas hetero-deficient PPARγ KO mice have a shorter lifespan [16]. It has also been reported that differences in adipokine secretion profiles affect lifespan. For instance, Otabe and colleagues revealed that liver-specific adiponectin transgenic mice are resistant to high-calorie diet-induced obesity and demonstrate an extended lifespan [17]. These reports support the notion that gene expression in WAT and adipokine secretion affect lifespan in rodents.

CR prevents age-induced adiposity by lowering plasma insulin and leptin concentration and raising adiponectin concentration [18,19], while also reducing the size of adipocytes in WAT (representative images are shown in Reference [20]). Therefore, we hypothesized that the beneficial effects of CR may be partially mediated by functional alterations in WAT. In the process of testing this hypothesis, we identified the sterol regulatory element-binding protein 1c (SREBP-1c), a master transcriptional regulator of lipogenic gene expression, as a mediator of CR. In this review, we discuss the SREBP-1c-dependent mechanisms of CR-induced metabolic alterations (metabolic remodeling) in WAT, principally with reference to our previous studies.

## 2. Identification of SREBP-1c as a Novel Mediator of CR

CR animals share many characteristics, such as smaller body size and lower plasma insulin concentration, with long-lived dwarf mice, which demonstrate suppression of GH/IGF-1 signaling [21,22]. This observation implies that the GH/IGF-1 axis and its related signaling pathway plays an important role in the effects of CR. In fact, it has been reported that GH receptor/GH-binding protein-deficient mice fail to exhibit the improvement in insulin sensitivity and extension in lifespan normally induced by CR, supporting the dependency of the effects of CR on the GH/IGF-1 axis [23]. IGF-1 promotes the nuclear export of forkhead box O (FOXO) transcription factors via phosphorylation of AKT [24,25]. Therefore, the suppression of GH/IGF-1 signaling transcriptionally upregulates the expression of FOXO-activated genes. Of the members of the FOXO family, FOXO1 and 3 have been shown to be involved in the anti-tumor and life-prolonging effects of CR, respectively, using genetically deficient mice [26,27]. However, CR further extends longevity in several long-lived animal models with suppressed GH/IGF-1 signaling, including Ames dwarf mice and heterozygous transgenic dwarf rats bearing an anti-sense GH transgene [28,29]. Hence, the beneficial effects of CR may not be dependent solely on GH/IGF-1 signaling. This notion is supported by a hepatic gene expression profiling study which showed that CR alters the expression of many genes involved in lipid metabolism in a GH/IGF-1-indepedent manner [30]. With this in mind, we compared gene expression profiles in WAT among ad libitum-fed rats, CR rats, and transgenic dwarf rats bearing an anti-sense GH transgene, to identify any WAT genes demonstrating CR-induced differences in expression that are GH/IGF-1-independent. This microarray analysis revealed that CR upregulates the expression of several genes involved in fatty acid (FA) biosynthesis in a GH/IGF-1-independent manner [31]. Another comprehensive analysis has also shown that CR increases the expression of genes involved in FA biosynthesis and adipogenesis in rat WAT, consistent with our microarray data [32,33]. In addition, it has been demonstrated that CR prevents age-related decline in FA biosynthesis in WAT [19]. Such effects on the expression of FA biosynthesis genes are likely to be mediated by an effect on their master regulator. Thus, we focused further investigations on the SREBPs, as upstream regulators of genes involved in FA biosynthesis.

SREBPs are transcription factors belonging to the basic helix-loop-helix/leucine zipper family that are known to be master regulators of lipid metabolism and adipocyte differentiation. Three isoforms exist, SREBP-1a, SREBP-1c, and SREBP-2 [34,35], all of which are synthesized as long inactive precursors that are bound to the endoplasmic reticulum, processed, and transported to the Golgi apparatus by SREBP cleavage-activating protein (SCAP). Following this, the SREBP NH_2_-terminal domain, which has transcription-modifying activity (nuclear SREBP; nSREBP) is translocated to the nucleus following proteolytic cleavage by two proteases residing in the Golgi apparatus, site-1 protease (S1P) and site-2 protease (S2P) [34,35]. SREBP-1a and SREBP-1c are encoded by a single gene, and their transcripts are distinguished on the basis of alternative transcription start sites. SREBP-1a is a more potent transcription factor, because it includes exon 1a, which encodes a longer acidic transactivation segment than the first exon of SREBP-1c, and stimulates the expression of genes involved in FA and TG biosynthesis. However, SREBP-1c is a more specific activator of the transcription of genes involved in FA biosynthesis than SREBP-1a. SREBP-2 is encoded by a different gene and preferentially enhances the transcription of genes involved in cholesterol biosynthesis [34,35].

We compared our microarray data with the list of genes regulated by SREBP-1 and SREBP-2 reported by Horton and colleagues [36], and found that of the genes involved in lipid metabolism, SREBP-1-regulated genes in particular were upregulated by CR, whereas SREBP-2-regulated genes were not [31]. To validate this result, we measured the mRNA expression of *Srebp-1a*, *Srebp-1c*, *Srebp-2*, and the genes regulated by SREBP-1 or SREBP-2, such as fatty acid synthase (*Fasn*), acetyl coenzyme A carboxylase (*Acc*), squalene epoxidase (*Sqle*), and mevalonate kinase (*Mvk*), in WAT in CR rats. CR induced a more marked upregulation of *Srebp-1c* than either of the other two isoforms [31]. Moreover, in accordance with the microarray data, CR upregulated the expression of SREBP-1-regulated genes, but did not affect the expression of SREBP-2-regulated genes [31]. Therefore, we concluded that CR predominantly induces the expression of SREBP-1c-regulated genes, specifically FA biosynthesis genes, and that SREBP-1c is a novel mediator of the effects of CR in WAT.

Several previous studies have demonstrated the roles of SREBP-1c in vivo using genetically manipulated mice. Shimano and colleagues showed that liver-specific nSREBP-1c transgenic mice exhibit moderately higher liver TG than SREBP-1a transgenic mice [37]. Conversely, Shimomura and colleagues showed that transgenic mice that overexpress nSREBP-1c in adipose tissue display features of lipodystrophy, including very small adipocytes, a highly fatty liver, and insulin resistance [38]. These seemingly contradictory results may be explained by the expression of the constitutively active form of SREBP-1c having been driven by the aP2 promoter, an extremely potent promoter in WAT. These findings raised the following question: What are the roles of SREBP-1c in WAT when its expression is induced by CR? To address this question, we compared the CR-induced responses in SREBP-1c KO mice with those in wild-type (WT) mice.

Initially, we evaluated the effects of CR on the lifespan of SREBP-1c WT and KO mice. Of note, CR-induced lifespan extension was abolished in SREBP-1c KO mice [39]. Furthermore, in agreement with our microarray data, CR-upregulated proteins were involved in FA biosynthesis, such as FASN, ACC, ATP citrate lyase (ACLY), and malic enzyme-1 (ME-1), in WAT of WT mice, but not in SREBP-1c KO mice [39]. These results suggest that SREBP-1c plays an important role in the effects of CR through the enhancement of FA biosynthesis in WAT.

## 3. Another SREBP-1c-Dependent Mechanism in the Effects of CR: Mitochondrial Activation

Many previous studies have demonstrated a relationship between CR and mitochondrial function in various organisms. Lin and colleagues demonstrated that in yeast, CR increases mitochondrial respiration, and the deletion of cytochrome c abolished CR-induced lifespan extension [40]. Subsequently, it has been demonstrated that in flies, mice, rats, and humans, CR increases the abundance of mitochondrial DNA (mtDNA) and the expression of mitochondrial genes and proteins [41,42,43,44,45]. Conversely, Lanza and colleagues have reported that CR improves mitochondrial function without enhancing mitochondrial abundance in skeletal muscle [46]. In accordance with the former reports, our previous proteomic analysis of WAT from *ad libitum*-fed and CR rats suggested that CR upregulates several mitochondrial proteins, as well as those involved in FA biosynthesis [20]. This proteome profile was validated by the demonstration that mtDNA and the activity of mitochondrial enzymes, including citrate synthase (CS) and cytochrome c oxidase, is enhanced in WAT from CR rats [20]. The microarray data published by Linford and colleagues also shows CR-induced upregulation of genes involved in mitochondrial oxidative phosphorylation in WAT, consistent with our proteomic data [32]. Moreover, as stated above, two long-lived animal models, FIRKO and β/β mice, show enhanced mitochondrial biogenesis in WAT [14,15], implying that mitochondrial changes in WAT can impact lifespan. On the basis of these findings, we focused on mitochondrial biogenesis as an SREBP-1c-dependent mechanism and showed that CR-induced upregulation of mitochondrial proteins, such as translocase of outer mitochondrial membranes 20 kDa (TOM20), cytochrome c oxidase subunit 4 (COX4), and sirtuin 3 (SIRT3), mtDNA content, and CS activity were abolished in WAT of SREBP-1c KO mice [39].

PPARγ coactivator-1α (PGC-1α) is a master transcriptional cofactor for mitochondrial biogenesis [47]. PGC-1α was identified as a PPARγ-interacting protein that is expressed preferentially in brown adipose tissue, a key thermogenic tissue [48]. PGC-1α stimulates mitochondrial biogenesis and respiration by binding to and activating nuclear respiratory factor (NRF)-1 and NRF-2, which enhance the transcription of a wide range of mitochondrial genes [49,50,51]. In addition to this, PGC-1α induces the expression of transcription factor A mitochondria (TFAM), a mitochondrial transcriptional factor required for the replication and transcription of mtDNA [51]. It has been reported that PGC-1α plays an important role in CR-induced mitochondrial biogenesis [42,52]. In our study, CR also significantly upregulated the expression of *Pgc-1α* mRNA in WAT of SREBP-1c WT mice. However, interestingly, SREBP-1c KO mice did not exhibit CR-induced upregulation of *Pgc-1α* mRNA [39]. We also demonstrated that the binding of SREBP-1c to the PGC-1α promoter region, where two sterol regulatory elements are located, was probably an SREBP-1c-dependent mechanism involved in the transcriptional activation of PGC-1α [39]. These results suggest that CR enhances mitochondrial biogenesis through SREBP-1c-dependent upregulation of PGC1α. The activity of PGC-1α is known to be regulated via its deacetylation by sirtuin 1 (SIRT1), a deacetylase induced during CR [53,54]. However, Rodgers and colleagues have shown that fasting- or CR-induced and SIRT1-regulated changes in hepatic PGC-1α expression cause upregulation of gluconeogenic, rather than mitochondrial genes [54]. However, we observed CR-induced upregulation of SIRT1 in WAT (unpublished data). Hence, despite no conclusive findings, CR-induced enhancement of PGC-1α expression and mitochondrial biogenesis in WAT might involve SIRT1, as well as SREBP-1c.

In addition, because CR generally suppresses oxidative stress, which is closely linked to mitochondrial function [55], we assessed two biomarkers of oxidative stress; the activity of aconitase, a mitochondrial enzyme vulnerable to oxidative stress [56], and the ratio of oxidized glutathione to reduced glutathione (GSSG/GSH), in WAT of SREBP-1c WT and KO mice. We found that CR significantly increases aconitase activity and reduces the GSSG/GSH ratio in SREBP-1c WT, but not KO mice [39]. Therefore, we conclude that SREBP-1c is required for CR-induced activation of mitochondria, including the enhancement of mitochondrial biogenesis and suppression of oxidative stress in WAT, but not in the liver, skeletal muscle, heart, or kidney.

## 4. Proposed Mechanisms for the CR-Induced Upregulation of SREBP-1c

In the above sections, we have described the CR-associated upregulation of SREBP-1c, which results in an increase in the expression of genes involved in FA biosynthesis. In this section, we discuss how CR might upregulate SREBP-1c and its downstream targets in WAT, using the findings from our chronological analysis of CR [57]. We measured the expression of FA biosynthesis factors and SREBP-1c in WAT of rats subjected to different durations of CR: 0.5 (short-term), 2, or 6 months (long-term). The mRNA and protein expression of FA biosynthesis factors was much higher in short-term and long-term CR, but only slightly higher after 2 months of CR, implying a V-shaped chronological effect of CR [57]. In contrast, SREBP-1c was upregulated at all stages of CR [57]. This discrepancy might be explained by differing transcriptional activity of SREBP-1c between short-term and long-term CR [57]. Next, we sought to evaluate the upstream regulators of SREBP-1c in these animals. It is well known that insulin signaling increases the expression of SREBP-1c [58,59], but in addition, leptin signaling downregulates the expression of SREBP-1c and FA biosynthesis genes [60]. Hence, we analyzed insulin and leptin signaling, and found that only short-term CR was associated with greater phosphorylation of nuclear AKT, a marker of insulin signaling [57]. Conversely, mRNA and protein expression of leptin was significantly lower, particularly following long-term CR [57]. These results suggest the phenotype of short-term CR may depend on insulin rather than leptin signaling, while that of long-term CR may depend on leptin rather than insulin signaling. Short-term CR can be regarded as an adaptive response to energy shortage, whereas long-term CR is usually used in experiments evaluating the beneficial effects of CR, including in our studies. Therefore, the mechanism underlying the CR-associated upregulation of SREBP-1c and FA biosynthesis genes in WAT might involve the predominant suppression of leptin signaling, rather than insulin signaling. Hereafter, further studies will be undertaken to determine whether there is a direct link between leptin signaling and CR-induced SREBP-1c.

## 5. Discussion

**Figure 1:**
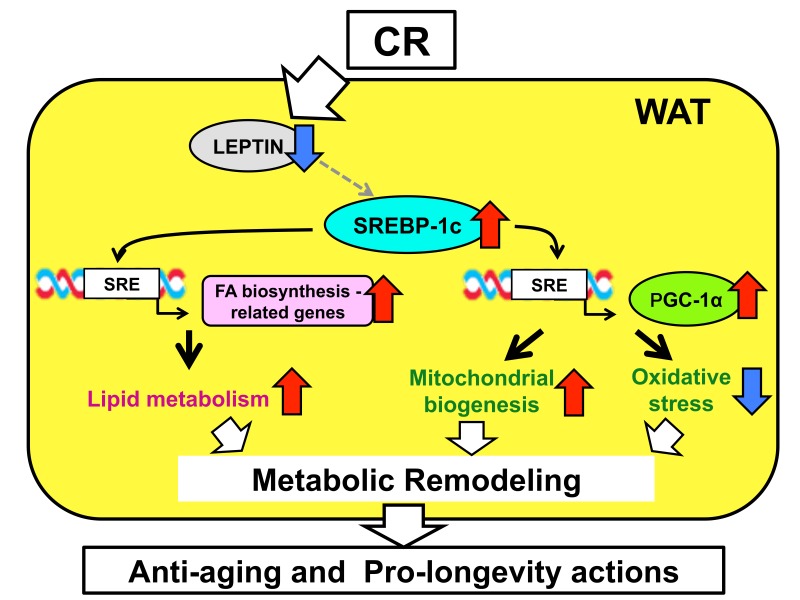
Summary of sterol regulatory element-binding protein 1c (SREBP-1c)-dependent mechanisms in the effects of caloric restriction (CR) in white adipose tissue (WAT). CR upregulates SREBP-1c, possibly via the suppression of leptin signaling. Upregulation of SREBP-1c enhances fatty acid (FA) biosynthesis and activates mitochondrial biogenesis and antioxidant capacity through the action of proliferator-activated receptor γ coactivator-1α (PGC-1α), by binding sterol regulatory element (SRE) in the promoter region of genes involved in these processes. This causes metabolic remodeling in WAT, which contributes to the anti-aging and pro-longevity effects of CR. Red arrows and blue arrows mean “upregulation” and “downregulation”, respectively.

The SREBP-1c-dependent mechanisms underpinning the effects of CR in WAT are presented in Figure 1. These comprise the enhancement of FA biosynthesis and the activation of mitochondrial function, and the possibility that the suppression of leptin signaling may also contribute to CR-induced SREBP-1c upregulation. Notably, these CR-induced effects were observed specifically in WAT, among the tissues we assessed [39]. For example, in liver, CR-induced upregulation of SREBP-1c occurred, but FA biosynthesis was not enhanced and mitochondria were not activated in our study [39]. One aspect of these findings was corroborated by another study showing that SREBP1 abundance and ME-1 activity are upregulated in WAT of CR rats [61]. Previous studies have shown that in liver, obesity is associated with higher expression of SREBP-1c but lower expression of PGC-1α [62,63,64]. Moreover, CR has been reported to prevent age-related induction of SREBP-1 and SREBP-2 in rat kidney, thereby reducing renal TG and cholesterol content [65]. These findings indicate that the response of SREBP-1c is likely to differ according to tissue type and circumstance. Furthermore, it has been reported that alterations to the microbiome are also associated with changes in the characteristics of WAT, such as browning (transformation of WAT into brown-like adipose tissue) and inflammation [66,67,68]. In addition, treatment with probiotics or prebiotics has been shown to downregulate high-fat diet or high cholesterol-induced SREBP-1c expression in liver [69,70]. As stated in the Introduction, several recently published articles have proposed a link between CR and the microbiome [8,71,72]. For example, Fabbino and colleagues demonstrated that the composition of the gut microbiome influences CR-induced metabolic alterations, including the improved insulin sensitivity and lower fat accumulation [72]. Thus, accumulating evidence suggests the possibility that the gut microbiome might contribute to CR-induced SREBP-1c upregulation and metabolic remodeling in WAT.

There are still several issues that require further investigation. For example, the significance of CR-induced enhancement of FA biosynthesis in WAT and the implications of the link between SREBP-1c and PGC-1α for the effects of CR. In general, lipids, represented by FA, are a more efficient source of energy than carbohydrates. A previous study revealed that CR significantly lowers the respiratory quotient and promotes FA oxidation, indicating that CR promotes whole-body lipid catabolism [73]. In addition, this study showed that CR enhances FA biosynthesis predominantly in subcutaneous and epididymal WAT, rather than in the liver [73]. Our recent study also demonstrated that CR-induced FA biosynthesis occurs in all three of the studied WAT depots: Retroperitoneal, epididymal, and subcutaneous [74]. These findings support the notion that WAT is a major source of lipids under CR conditions. Therefore, we hypothesize that CR can induce a shift in the substrate used to generate energy from carbohydrate to lipid through the upregulation of SREBP-1c in WAT.

As stated above, SREBP-1c was originally identified as a master transcriptional upregulator of FA biosynthesis genes, such as *Fasn*, *Acc*, and *Acly*, in response to nutrient status [34,35]. In addition to these genes, we found that *Pgc-1α* was also a direct target of SREBP-1c, and that SREBP-1c mediates CR-induced mitochondrial activation [39]. We explain these findings as follows. Citrates, produced from oxaloacetates by CS in mitochondria, are important intermediates in FA biosynthesis [75]. FA biosynthesis begins with the translocation of citrates from mitochondria to the cytosol. Subsequently, these citrates are converted into acetyl CoA by ACLY, and eventually into FA by ACC and FASN in the cytosol [76]. Furthermore, interestingly, previous experiments, mainly conducted in yeast, have demonstrated that mitochondria are able to synthesize FA [77]. Thus, despite having no direct evidence, we consider that SREBP-1c-dependent mitochondrial activation might constitute a component of the mechanism underlying CR-induced enhancement of FA biosynthesis.

Recently, the development of CR mimetic medicines for treating age-related diseases has been initiated, and this is actively ongoing. We believe that elucidating the molecular mechanisms of CR is essential for this process to succeed. The findings shown in this review demonstrate that CR enhances FA biosynthesis and activates mitochondrial function in WAT, resulting in metabolic remodeling. In addition, SREBP-1c and PGC-1α are identified as mediators of these effects of CR. Therefore, SREBP-1c and PGC-1α could be novel molecular targets of a CR mimetic, and further analysis of the mechanisms involving these molecules could lead to advances in the development of CR mimetic medicines.
